# Average power and burst analysis revealed complementary information on drug-related changes of motor performance in Parkinson’s disease

**DOI:** 10.1038/s41531-023-00540-x

**Published:** 2023-06-16

**Authors:** Flavie Torrecillos, Shenghong He, Andrea A. Kühn, Huiling Tan

**Affiliations:** 1 Medical Research Council Brain Network Dynamics Unit, University of Oxford, Oxford, UK; 2 Nuffield Department of Clinical Neurosciences, University of Oxford, Oxford, UK; 3 Department of Neurology, Charitè, Universitätsmedizin, Berlin, Germany

**Keywords:** Parkinson's disease, Basal ganglia

## Abstract

In patients with Parkinson’s disease (PD), suppression of beta and increase in gamma oscillations in the subthalamic nucleus (STN) have been associated with both levodopa treatment and motor functions. Recent results suggest that modulation of the temporal dynamics of theses oscillations (bursting activity) might contain more information about pathological states and behaviour than their average power. Here we directly compared the information provided by power and burst analyses about the drug-related changes in STN activities and their impact on motor performance within PD patients. STN local field potential (LFP) signals were recorded from externalized patients performing self-paced movements ON and OFF levodopa. When normalised across medication states, both power and burst analyses showed an increase in low-beta oscillations in the dopamine-depleted state during rest. When normalised within-medication state, both analyses revealed that levodopa increased movement-related modulation in the alpha and low-gamma bands, with higher gamma activity around movement predicting faster reaches. Finally, burst analyses helped to reveal opposite drug-related changes in low- and high-beta frequency bands, and identified additional within-patient relationships between high-beta bursting and movement performance. Our findings suggest that although power and burst analyses share a lot in common they also provide complementary information on how STN-LFP activity is associated with motor performance, and how levodopa treatment may modify these relationships in a way that helps explain drug-related changes in motor performance. Different ways of normalisation in the power analysis can reveal different information. Similarly, the burst analysis is sensitive to how the threshold is defined – either for separate medication conditions separately, or across pooled conditions. In addition, the burst interpretation has far-reaching implications about the nature of neural oscillations – whether the oscillations happen as isolated burst-events or are they sustained phenomena with dynamic amplitude variations? This can be different for different frequency bands, and different for different medication states even for the same frequency band.

## Introduction

Deep brain stimulation (DBS) is an established and effective treatment to manage the symptoms of advanced Parkinson’s disease (PD), which also offers a unique opportunity to record subcortical electrophysiological activity in human participants^1,2^. The local field potential (LFP) signals recorded from the depth electrodes reflect the underlying activity of neuronal populations and hence provide direct insight into basal ganglia function. Many studies have investigated how the power of different oscillatory activities in the subthalamic nucleus (STN) are broadly associated with motor functions. For example, beta (13–30 Hz) and gamma (31–90 Hz) bands have gained most attention due to their robust movement reactivity. It has been shown that the latency of beta power desynchronization in the STN correlates with reaction time across and within subjects^3,4^. The percentage changes in the power of the beta and gamma oscillations correlated with movement vigour^5–7^ and other kinematic parameters^8–10^. In addition, averaged beta and gamma powers in the STN have been found to correlate with hand bradykinesia during sustained gripping^11^ and repetitive tapping^12^ in PD.

Most previous studies mentioned above quantified the power of oscillatory activities during different time windows averaged across trials. More recently, focus on the temporal dynamics of LFP signal has revealed that beta and gamma oscillations may consist of transient bursting episodes that last a few cycles rather than sustained oscillatory activities^13,14^. This has led to the speculation that the modulation of burst characteristics such as burst duration and rate, which reflect the changes in the temporal dynamic of the power of targeted oscillations, might contain more information about pathological states and behaviour^15–18^. For STN LFPs, slowing of repetitive movements have been associated with increased burst duration and percentage of time spent in beta bursts^19,20^ and with a decrease in gamma burst rate within PD patients^10^. Two further studies have focussed on the motor effects of trial-by-trial changes in beta burst characteristics before cued movement onset in PD patients, demonstrating that STN beta bursts in specific time windows can reduce the velocity of externally-triggered ballistic movements, an effect that can be amplified by the presence of longer duration and multiple bursts, and ones that overlap in time across the two STNs^21,22^.

Interpreting frequency-specific patterns of neural activity as transient bursts of isolated events rather than as rhythmically sustained oscillations has potentially far-reaching theoretical implications^14,23^. It challenges models of how such ongoing oscillations serve to route information flexibly and also questions models proposing that oscillations are generated through ongoing recurrent excitation and inhibition^14^. Computational modelling has shown that transient beta events in the motor cortex could emerge from the integration of synchronous bursts of excitatory synaptic drive targeting proximal and distal dendrites of pyramidal neurons^24^. Meanwhile, several recurrent circuits in the cortico-basal ganglia-thalamic network have been identified to be able to generate, propagate and maintain excessive beta oscillations in PD^25–28^.

Therefore, we argue that the STN LFP signals reflect a mixture of sustained level of rhythmic synchrony and transient bursting events. This raises the possibility that both features of the subcortical oscillatory activity – sustained oscillation which can be quantified by power and transient bursting events which are better quantified by bursting parameters – might provide complementary information about the pathophysiology and motor performance within PD patients^29^. Here, we test this hypothesis through a direct comparison of the effect of mean power and bursting patterns on how they differentiate dopamine states and how they can predict motor performance on trial-by-trial basis in PD. In addition, bursts in neural time series are usually identified by applying a simple binary decision threshold based on the pooled distribution of amplitude values. When using burst analysis to compare the neural signals recorded when the patients are ON vs OFF dopaminergic medication to understand the pathophysiology of PD, decision has to be made on either defining the burst threshold on the single condition (ON or OFF) or the combined data. Using different thresholds on the same time series does not alter its dynamics, it only alters how we binarise the time series and define bursts. Therefore, we also investigated how different thresholding methods (within single medication condition separately vs one common threshold based on combined data) change the results.

## Results

### PD patients perform faster self-paced movements ON medication

**Fig. 1:**
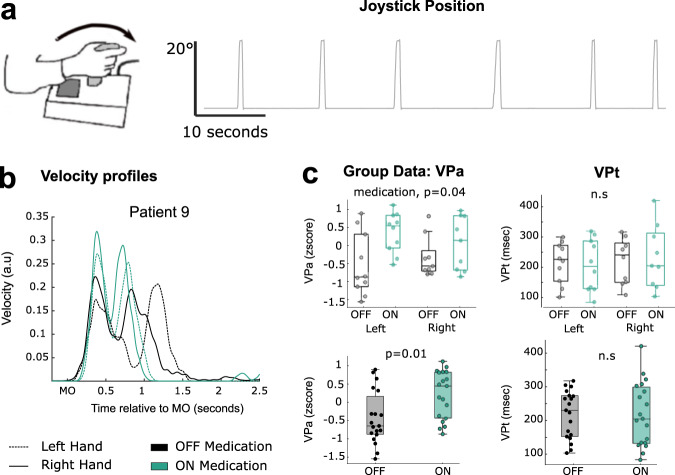
Experimental Protocol and behavioural results. **a** Patients performed stereotyped self-paced movements of a hand-held joystick repeated approximately every 10–20 s. **b** Averaged velocity profiles for a representative patient in the four conditions. **c** Averaged velocity peak amplitudes and latencies at the group level, in the four conditions (top) and when movements of the left and right hands are grouped together (bottom). The circles and dots indicate data from different individuals. In each box plot, the central line indicates the median, and the bottom and top edges of the box indicate the 25th and 75th percentiles across all recorded samples, respectively. The whiskers extend to the most extreme data points (minima and maxima) not considered outliers. VPa: Velocity peak; VPt: time from movement onset to velocity peak. Each dot represents one STN. n.s: not significant; msec: milliseconds.

In the current study, PD patients were instructed to perform ballistic self-paced joystick movements with their left or right hands both OFF and ON medication (see Methods, Fig. 1a). The individual velocity profiles were computed for each of the four conditions separately (OFF-left hand, OFF-right hand, ON-left hand, and ON-right hand) and normalized by z-scoring across hands and states within a subject to facilitate group analysis. As can be seen in Fig. 1b for a representative patient, there are two peaks in the velocity profiles corresponding to the forward and backward movement of the joystick respectively. Velocity peaks of the forward movements were identified on each single trial and both their amplitude (VPa) and latency (VPt) were extracted. The effects of both hand (Left / Right) and medication (ON /OFF), as well as their interaction, were then tested on VPa and VPt by applying linear-mixed effect models. The results revealed a significant effect of medication on mean VPa (Fig. 1c, *b* = 1.41 ± 0.11, t_(34)_ = 2.13, *p* = 0.04), but no effect of hand (*b* = 0.72 ± 0.12, t_(34)_ = 1.1, *p* = 0.29) or interaction between hand and medication (*b* = −0.51 ± 0.07, t_(34)_ = −1.2, *p* = 0.24). No significant effect was observed on VPt (hand effect: *p* = 0.45; Medication: *p* = 0.92; Interaction: *p* = 0.35). These results suggest that PD patients performed faster movements ON medication, independently of the hand used to hold the joystick.

Based on these behavioural results, we decided to focus our analysis on VPa, and to group the movements performed with left and right hands together treating each hand and the contralateral STN as independent samples. In this way we considered a group of 19 STNs OFF medication (sub5 performed the task with only the left hand) and 17 STNs ON medication (recordings ON medication of sub3 were compromised). The comparison of mean VPa between the two medication states confirmed the results described above, with faster forward movements performed ON than OFF medication (Fig. 1c, paired t-tests, t_(18)_ = −2.89, *p* = 0.01).

### Modulation of subthalamic activities by dopaminergic medication at rest

**Fig. 2:**
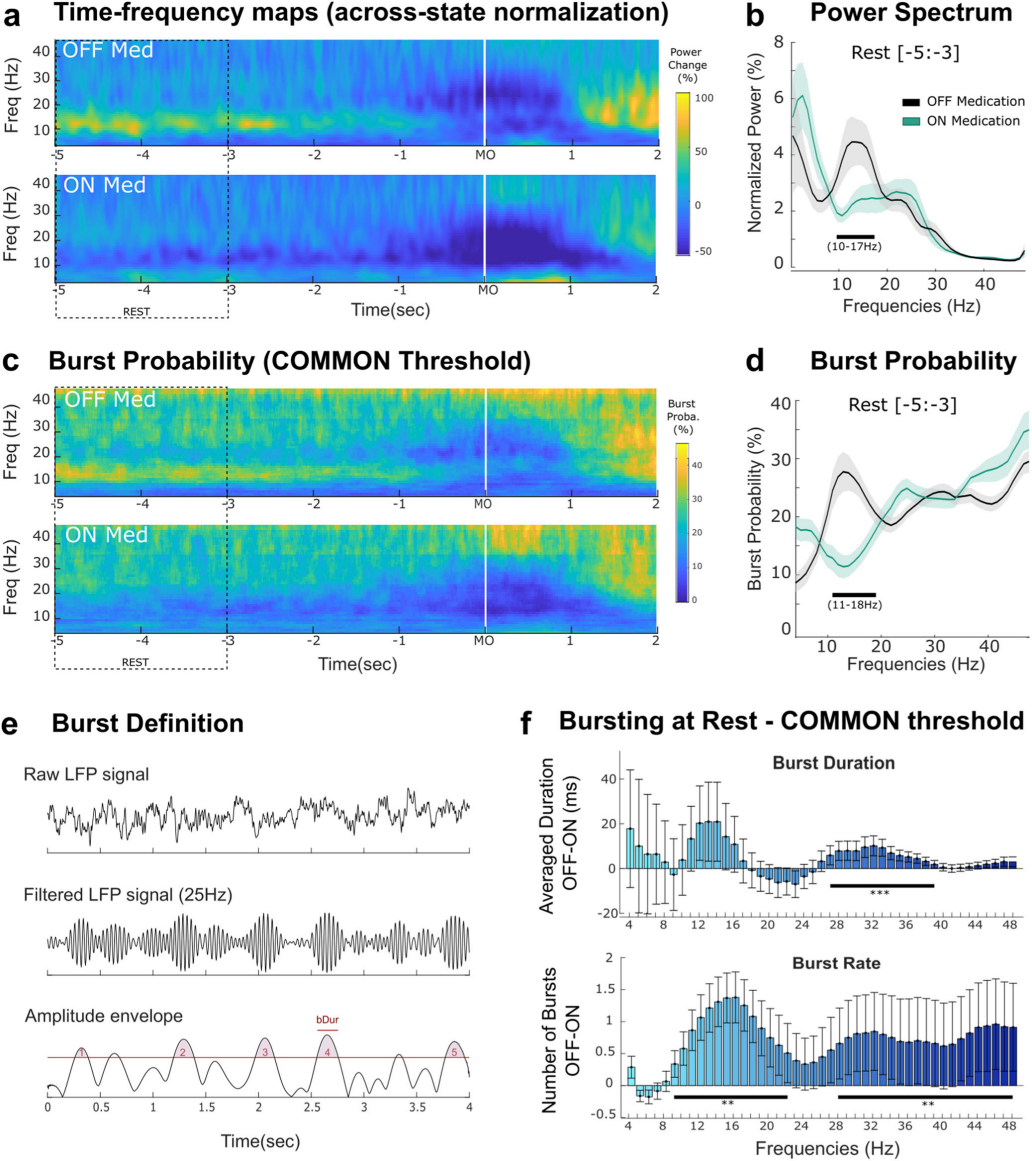
Power and burst analysis with across-state normalization revealed drug-related modulation in the low-beta frequency band in STN at rest. **a** Averaged time frequency plots aligned to the movement onset (MO) both OFF and ON medication. To visualize the effect of medication, an across-state normalization is used (average OFF-ON), similar to a common burst threshold. **b** Averaged power spectral densities for both medication states at rest (from −5 to −3 seconds before MO, see dashed box in plot **a**. Power values are normalized to the average power across both medication states in the 1–100 Hz to allow group average (see methods). The solid line and shaded area indicate mean values +/− sem. Cluster based-permutation test revealed a significant cluster from 10 to 17 Hz (black bar). **c** Averaged burst probability over time for all frequencies from 4 to 48 Hz. Burst probabilities are computed across trials based on a common burst threshold and then averaged across all STNs. **d** Averaged burst probabilities (*n* = 19 STNs) at rest. The solid lines and shaded areas indicate the mean +/− sem across all STNs, respectively. Cluster based-permutation test revealed a significant cluster from 11–18 Hz (black bar). **e** Schematic of burst definition. Raw LFP signals are filtered at a specific frequency and a threshold is applied to the amplitude envelope (75^th^ percentile, see text). In this example 5 bursts are identified. **f** Burst analysis revealed additional changes in the low-gamma frequency bands between OFF and ON medications. The vertical bar and whiskers indicate the mean +/− sem across all STNs. Frequencies at which the paired comparison between OFF and ON is significant are indicated with the horizontal black bars (cluster-based permutation tests, *p < 0.05, **p < 0.01, ***p < 0.001, after FDR correction).

One important pathological feature of the STN-LFP activity in the dopamine-depleted state is its excessive synchrony in the beta frequency band, clearly visible on the time-frequency maps when normalizing the signals across states (Fig. 2a). The comparison of the power spectral densities (PSDs) at rest with a cluster-permutation test allowed us to identify a range from 10 to 17 Hz where power was significantly higher in the OFF compared to the ON state (Fig. 2b, *p* = 0.03).

In addition to the effect on STN beta power, dopaminergic treatment has been suggested to also change the temporal dynamics of beta power, reflected in the relative distribution of beta bursts, which become longer when OFF medication compared to ON^16^. To allow comparison with this previous study we first defined bursts based on a common threshold across both medication states (Fig. 2e). The bursts analysis revealed similar results as the average power, with increased oscillatory activities in the low beta frequency band at rest, characterized by an increase in burst probability across trials (Fig. 2c, d, cluster from 11 to 18 Hz, *p* = 0.05), an increase in the number of bursts per trial (burst rate, Fig. 2e, f, cluster from 9 to 22 Hz, *p* = 0.007). In addition, the comparison of burst rate and duration between the two medication states revealed that bursts during OFF medication were longer than ON medication for the frequencies ranging from 27 to 39 Hz (*p* < 0.001) and occurred at a higher rate between 28 and 48 Hz (Fig. 2e, f, *p* = 0.019).

**Fig. 3:**
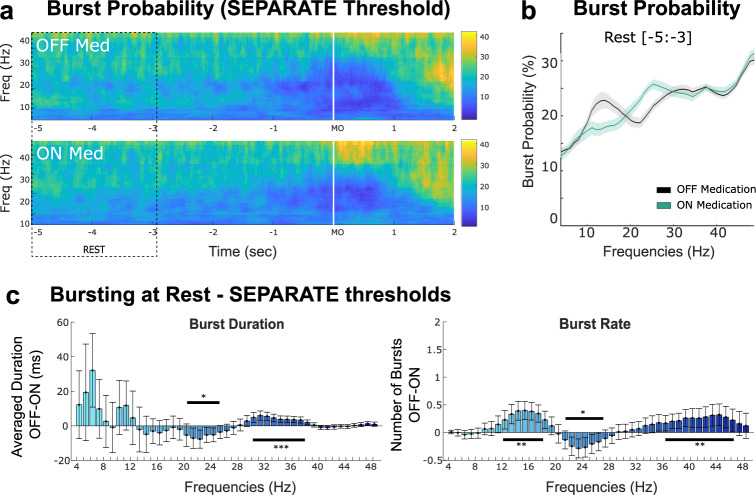
Burst analysis with separate thresholds revealed difference in drug-related modulation for the low-beta vs high-beta frequency bands in STN at rest. **a** Averaged burst probability over time for all frequencies from 4 to 48 Hz. Burst probabilities are computed across trials based on two separate thresholds defined within each medication state, and then averaged across all STNs. **b** Averaged burst probabilities (*n* = 19 STNs) at rest (from −5 to −3 s before MO, see dashed box on A.). The solid lines and shaded areas indicate mean +/− sem. **c** Differences in burst duration and burst rate between OFF and ON medication states at rest for all frequencies from 4 to 48 Hz. The vertical bars and whiskers indicate the mean +/− sem across all STNs. Frequencies at which the paired comparison between OFF and ON is significant are indicated with the horizontal black bar (cluster-based permutation tests, *p < 0.05, **p < 0.01, ***p < 0.001, after FDR correction).

To control for the influence of the medication-related modulation of mean power in the burst analysis, we re-defined bursts using separate thresholds, which helps emphasize the within-state dynamic changes (Fig. 3). This revealed additional medication-related changes in the high-beta frequency range, with shorter burst duration (20–25 Hz, *p* = 0.02) and smaller number of bursts (21–27 Hz, *p* = 0.03) when OFF medication compared with ON medication, opposite to the direction of change in burst rate in low-beta frequency band (Fig. 3c, 12–18 Hz, *p* = 0.004). Note that the modulations observed in the low gamma band with a common threshold were still present (duration, cluster from 30 to 38 Hz, *p* < 0.001; rate, cluster from 34 to 46 Hz, *p* = 0.003).

All together, these results suggest that average power, burst duration and rate are modulated by levodopa, in a frequency-specific way. Burst analysis reveal a complementary modulation of the LFP activity in the gamma band that was not observed in the averaged power. In addition, the results of these analysis in the beta band are strongly dependent on how bursts are defined, and separate thresholds are able to identify opposite modulation of bursts in the two beta sub-bands.

### Modulation of subthalamic activities before and during voluntary movements

As already observed in Fig. 2a, c, power and burst probabilities were modulated by movements, with a clear desynchronization during movement spanning the alpha and beta frequency ranges. To evaluate the effect of medication on this movement-related profile, power time courses were defined in four predefined frequency bands and normalized across medication state or within medication states (see Methods).

**Fig. 4:**
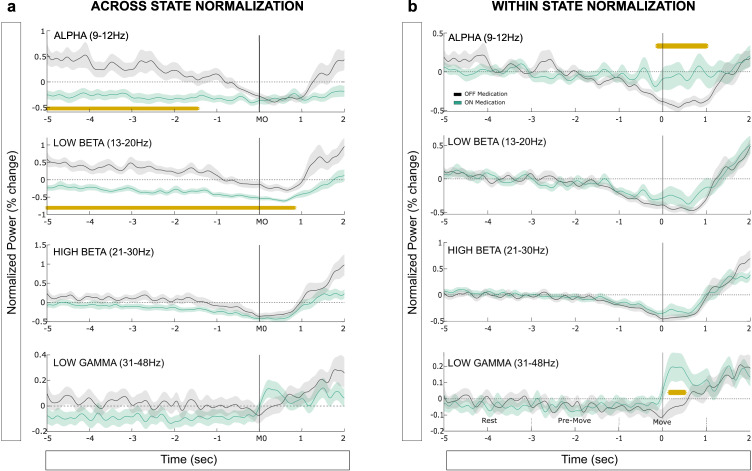
The two normalizations revealed different information about how movement-related modulation of STN power was affected by medication in 4 frequency bands. **a** Medication-related changes in averaged power for the 4 pre-defined frequency bands. Power values are normalized against the mean value in that frequency band across the two medication states (average OFF and ON). The time points at which the contrast between OFF and ON states is significant are indicated with a yellow bar (significant clusters). **b** Same as in plot (**a**) but with power values normalized separately within each medication state against the mean value in each frequency band. The time points at which the contrast between OFF and ON states is significant are indicated with a yellow bar. The solid lines and shaded areas indicate mean +/− sem. (see text for details of the statistical tests).

**Fig. 5:**
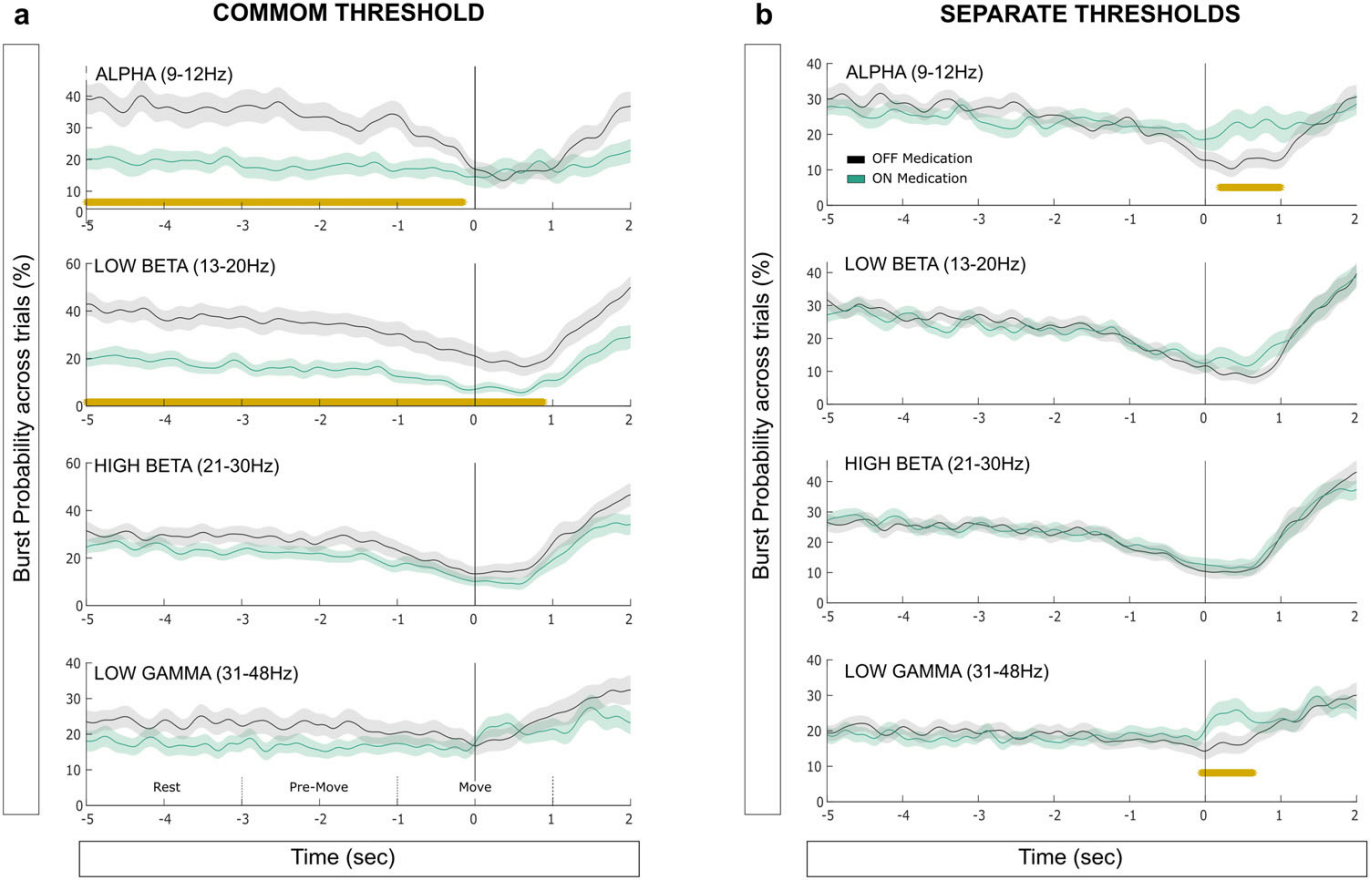
The two burst thresholds revealed different information on how movement-related modulation of the burst characteristics were affected by the medication. **a** Medication-related changes in burst probability for the 4 pre-defined frequency bands. Bursts are defined with a common threshold (OFF-ON) and the probability of bursting is defined across trials as percentage. The time points at which the contrast between OFF and ON states is significant are indicated with a yellow bar. **b** Same as in plot (**a**) but with separate burst thresholds used within each medication state. The solid lines and shaded areas indicate mean +/− sem. The time points at which the contrast between OFF and ON states is significant are indicated with a yellow bar. (see text for details of the statistical tests).

When the power was normalised across medication states, we observed higher power in the absence of levodopa in the alpha band from 5 to 1.45 s before the movement onset (*p* = 0.005, Fig. 4a) and until approximately the end of the movement for the low beta power (from 5 to 0.83 s, *p* < 0.001, Fig. 4a). For burst analysis, the use of a common threshold revealed similar effects on the probability of alpha (cluster from −5 to −0.15 s, *p* = 0.01, Fig. 5a) and low beta bursts across trials (cluster from −5 to 0.89 s, *p* = 0.003, Fig. 5a). Note that the effect of medication on the alpha bursts was however prolonged compared to alpha power, and revealed a difference until just before movement onset (0.15 s before) in contrast to 1.45 s with power.

When the power was normalised within-state and bursts defined using separate thresholds, different effects of the medication were revealed (Figs. 4b, 5b). While the previous pre-movement modulation disappeared in both alpha and low beta band, an increase of power was observed with levodopa during movement in the alpha (from −0.13 to 1 s, *p* = 0.002) and low gamma bands (from 0.15 to 0.5 s, *p* = 0.012). Again, similar results were reported when looking at burst probability with an effect in both alpha (from 0.19 to 1 s, *p* = 0.043) and low gamma bands (from −0.06 to 0.64 s, *p* = 0.033).

**Fig. 6:**
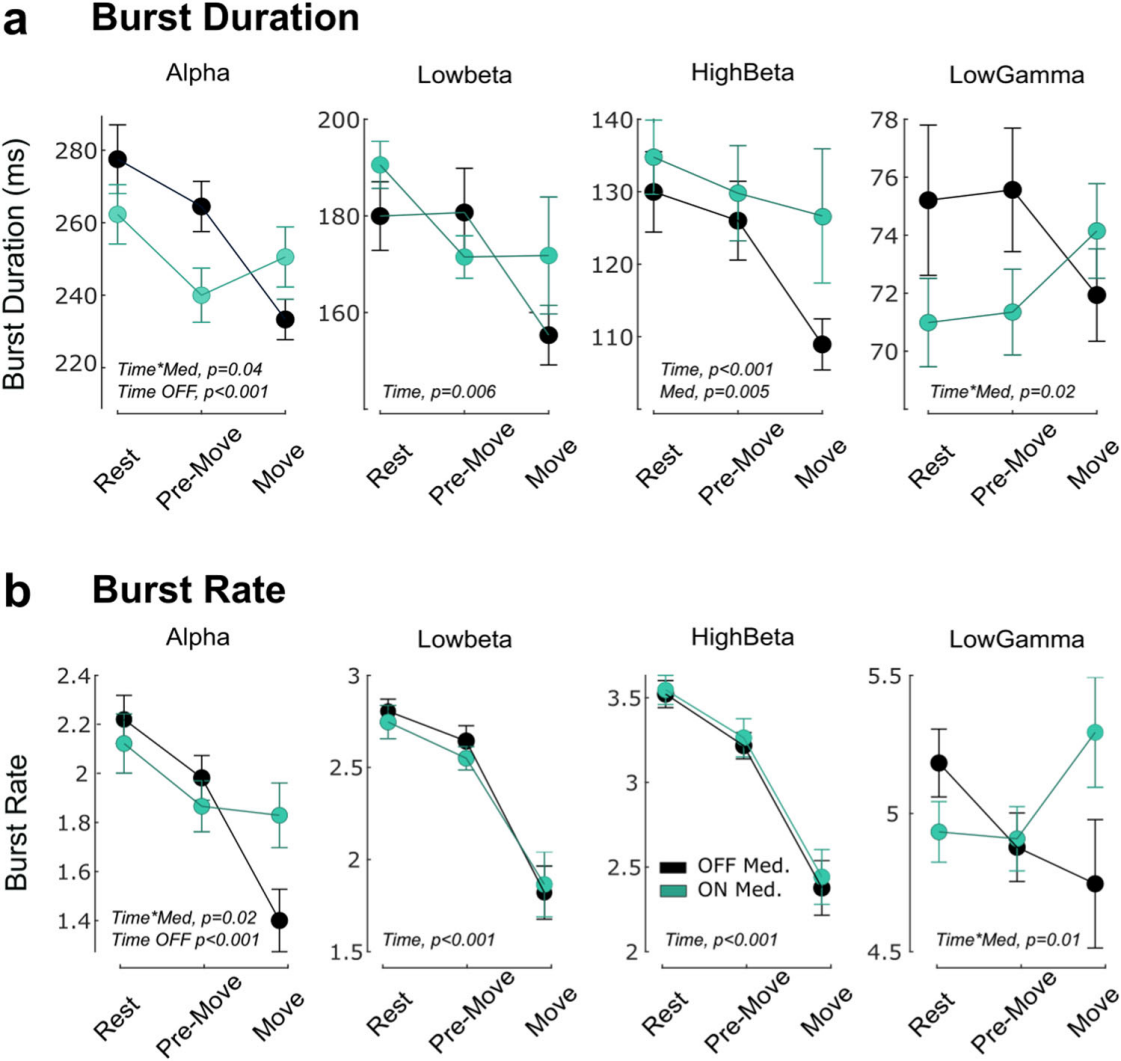
Movement-related modulation of burst duration and rate, and the interaction with medication in 4 frequency bands. **a** Changes in the mean burst durations between the three time-windows and the two medication states when bursts are defined with a common threshold. **b** Same as in plot (**a**) for burst rate. In both (**a**) and (**b**), dots and error bars indicate mean +/− sem, with black and green for OFF and ON medication, respectively. The significant linear-mixed effects models are indicated on the figure. See text for further details.

We then asked whether these new medication-related changes in bursting activity were explained by a change in the rate or duration of bursts. Linear mixed effect models were used to test the effect of time (3 windows) and medication (OFF/ON) and their interaction on both burst rate and mean burst duration (Fig. 6). The results showed the preparation and execution of movement were associated with a modulation of both burst rate and duration, but differently for each frequency band and medication state. First, gradual reductions of burst duration and rate were observed from rest to movement execution in the low beta (duration: *b* = −10.9 ± 3.8, t_(111)_ = −2.8, *p* = 0.005, rate: *b* = −0.5 ± 0.06, t_(111)_ = −4.8, *p* < 0.001) and high beta (duration: *b* = −7.3 ± 1.9, t_(111)_ = −3.9, *p* < 0.001, rate: *b* = −0.6 ± 0.05, t_(111)_ = −10.3, *p* < 0.001) bands, independently of the medication state. In contrast, modulation of both alpha and gamma band bursts were conditioned by the medication state (significant interactions). While OFF levodopa rate and duration of alpha bursts were reduced when approaching movement (duration: *b* = −22.1 ± 4.6, t_(55)_ = −4.8, *p* < 0.001; rate: *b* = −0.4 ± 0.07, t_(55)_ = −5.5, *p* < 0.001), no significant modulation were observed ON medication (duration: *b* = −5.9 ± 5.0, t_(55)_ = −1.2, *p* = 0.025; rate: *b* = −0.1 ± 0.08, t_(55)_ = −1.8, *p* = 0.08). In the gamma band, interaction effects were however not followed by significant effects within either of the medication state. Finally, note that high-beta bursts were longer ON than OFF also around movement, in line with the observations made at rest (Fig. 3).

All together these results confirmed that both power and bursting activities are modulated in preparation to the execution of a voluntary movement. Normalising power or defining bursts within the medication state helped to reveal increased activities in the alpha and gamma frequency bands during movements when on dopaminergic medication. In that medication state, the modulation of bursts duration and rate seem specifically tuned to the beta band, whereas they expand to the adjacent frequency bands in the absence of levodopa.

### Power and burst analysis allow the prediction of following motor performance

**Fig. 7:**
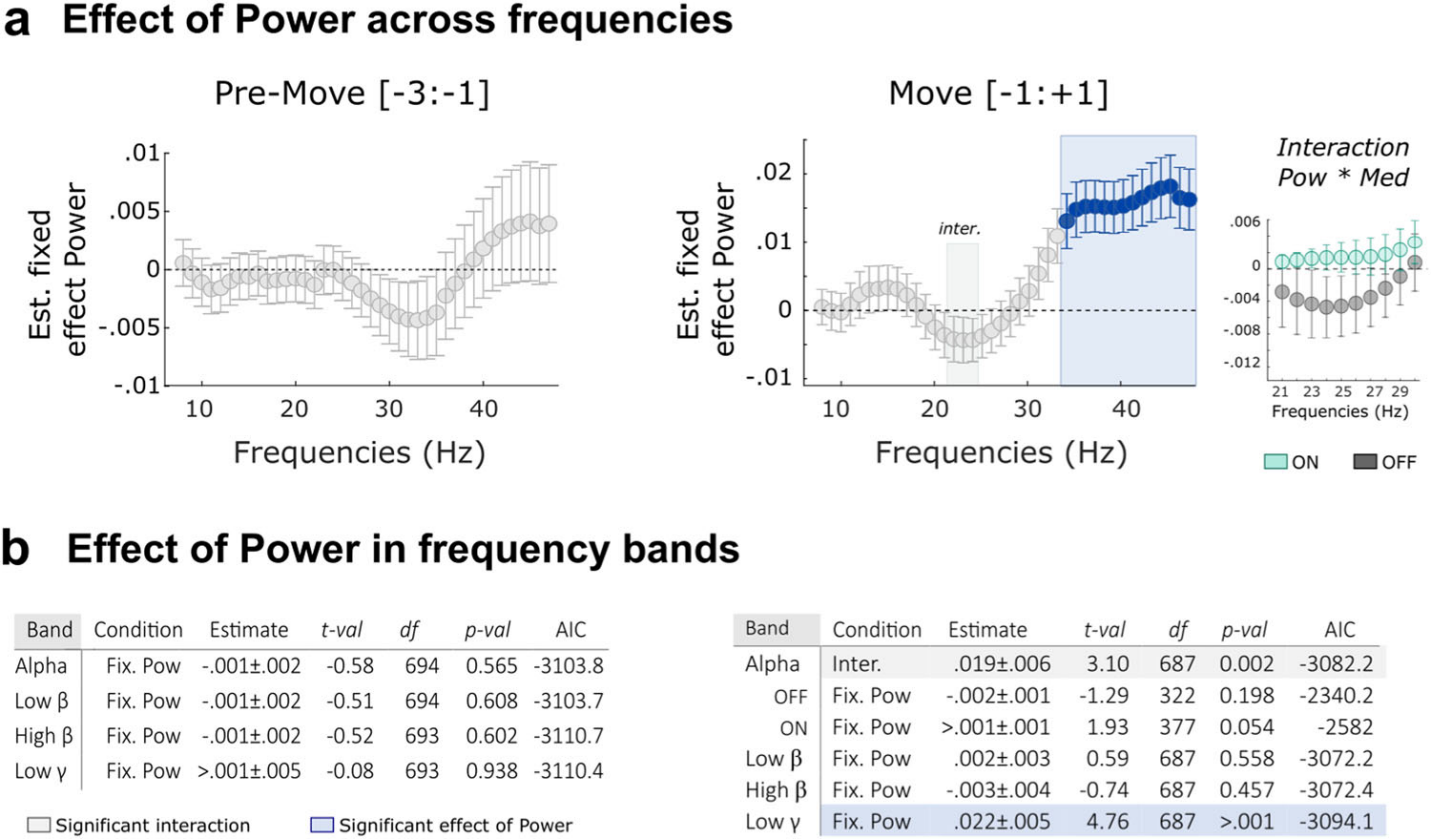
Trial-to-trial relationship between movement velocity and average STN power in different frequency bands. **a** Linear-mixed effect models revealed that higher power in the low gamma frequency band (33–48) around movement onset (−1:1 s) predicted higher peak velocity (Likelihood ratio test, *p* < 0.05 after FDR correction, and highlighted with a blue shading). The model also identified an interaction between medication and averaged power in the high-beta frequency band (22–24 Hz when each frequency with 1 Hz resolution were considered separately, highlighted in grey). Follow-up LMEs are run around these frequencies to test the effect of Power within each medication state (see *right*). The circles and error bars indicate the estimated effect +/− SE. **b** Details of LME modelling results when power are averaged within the 4 pre-defined frequency bands (same as Figs. 4, 5). The significant models after FDR correction are highlighted in blue (fixed effect) or grey (interaction). Models with significant interactions are followed by within-state models. Df: degree of freedom AIC: Akaike information criterion.

In parallel to its sensitivity to medication, subthalamic LFP activity has been shown to correlate with motor symptoms and motor performance in PD. We then asked whether both power and burst parameters could be related to motor performance on a trial-by-trial basis and if so how. We first started by considering averaged power for all frequencies from 8 to 48 Hz in the pre-Move and Move windows. Note that for this analysis, the within-state normalization was used to focus on the movement-related dynamics. Linear-mixed effect models were applied to predict the peak velocity of each individual trial treating average power of each frequency at different time windows, medication states and their interaction as fixed effects. The results revealed that faster movements were associated with higher power in every frequency from 33 to 48 Hz (corrected *p* < 0.05 for all the 16 frequencies) in the time window around movements (−1 to 1 s, Fig. 7a). In addition, a significant interaction between power and medication was observed for the frequencies 22–24 Hz. This interaction was related to the opposite effects that high beta power had on movement velocity in the two medication states (positive estimates ON but negative OFF).

The linear-mixed effects modelling analysis were then repeated with power averaged in the four previously defined frequency bands (Fig. 7b). This confirmed the effects observed in the Move window with higher gamma power around movement associated with faster movements (see Fig. 7b for the details of the LME). This relationship was consistent across individual STNs (14/19 STNs). An interaction was also revealed but this time in the alpha frequency band, followed by a positive trend in the ON medication state (*b* > −0.001 ± 0.001, t_(377)_ = 1.93, *p* = 0.054) that would suggest that higher alpha power around movement onset is associated with faster movements.

**Fig. 8:**
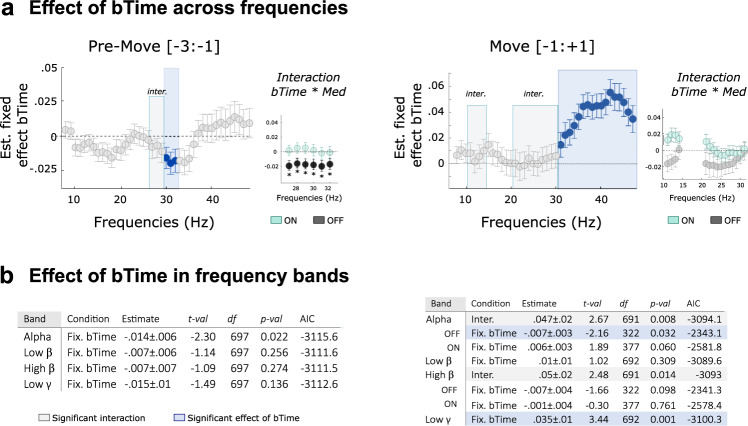
Trial-to-trial relationship between movement velocity and time spent in bursts for different frequency bands. **a** Models with time spent in bursts as predictors showed very similar results as those for average power: most consistently, time spent in gamma bursts around movements positively predicted higher peak velocity (Likelihood ratio test, *p* < 0.05, FDR corrected for multiple comparison, high-lighted in blue). Interaction between medication and time in bursts was also observed in the high beta frequency ranges (grey shading) and were followed by significant models OFF state (pre-move, black dots and *). The circles and error bars indicate the estimated effect +/− SE. **b** Details of the LME modelling results when burst are defined in predefined frequency bands. The significant models after FDR correction are highlighted in blue (fixed effect) or grey (interaction). Df: degree of freedom AIC: Akaike information criterion.

We next used the same approach to evaluate the role of the burst parameters on movement velocity within subject. To combine both the duration and the rate of burst in a single predictor we used the time spent in burst, expressed as percentage. To focus on the movement dynamic, only bursts defined with a separate threshold were considered. In contrast to the analysis with average power, significant associations were identified in the pre-Move time window, with faster movements associated with less time spent in bursts in a 27–32 Hz frequency range, especially in the OFF medication state (Fig. 8a, corrected p < 0.05 for all 6 frequencies). However, this was not confirmed when considering pre-defined frequency bands (Fig. 7b) probably due to the narrowness of the effect and its overlap with both the high-beta and low gamma range. Note that a trend was observed in the alpha band but did not survive the correction for multiple comparison. Finally, around movement, the results revealed a similar pattern as with averaged power with a positive and strong effect observed between movement velocity and the time spent in gamma bursts (from 31 to 48 Hz, confirmed by taking bursts in the low gamma range, *b* = 0.22 ± 0.005, t_(687)_ = 4.8, *p* < 0.001). In addition, two clusters with significant interactions were revealed from 11 to 14 Hz and 21 to 31 Hz, corresponding to the alpha and high beta band respectively (Fig. 7a, b, alpha band: *b* = 0.05 ± 0.02, t_(691)_ = 2.7, *p* = 0.008, high-beta band: *b* = 0.05 ± 0.02, t_(691)_ = 2.5, *p* = 0.014) These interactions are explained by opposite relationships observed between movement velocity and bursting activity when OFF versus ON medication (see Fig. 8a right). Follow-up tests showed that alpha bursts impact on movement velocity but only in the OFF state (*b* = −0.007 ± 0.003, t_(322)_ = −2.2, *p* = 0.03). Interestingly this corresponds to the state where alpha power is desynchronized and bursts duration and rate are reduced (see above, Figs. 4–6). Note that all the above relationships were found when using the burst rate as predictor, but not the mean burst duration.

All together, these results showed that both averaged power and bursting parameters in different frequency bands can have an impact upon motor performance. Both the power and burst analyses identified that the increase of low gamma band activity (33–48 Hz) during movements predicts higher peak velocity, with no interaction between medication and the power or time in burst (see Figs. 7, 8). In addition, both the power and burst analyses identified interaction between medication and the high beta band activity (21–30 Hz) during movement in predicting peak velocity. Therefore, we have included the trial-by-trial absolute power and the ‘time in burst’ in the same model (details in the supplementary material). The effect of burst properties survived with absolute power as a covariate. However, the effect of the absolute power had opposite directions (details in the supplementary material). The ‘time in burst’ and ‘absolute power’ were highly correlated especially when the absolute power was calculated over short time windows on trial-by-trial basis, which was confirmed by the high variance inflation factors (VIF) between time in the burst and average power (5.18 and 6.47 for low gamma and high beta respectively). The collinearity in the independent variables can make the multi-variate models less reliable and more difficult to interpret.

## Discussion

In this study we compared how STN average power and bursting parameters were modulated by both anti-parkinsonian medication and the execution of self-paced movements, for different frequencies. First, we found that both power and bursts analyses were able to confirm the exaggerated synchrony in the low-beta frequency band typically observed at rest in the dopamine depleted state, but only the latter revealed a modulation in the low gamma range. Second, we showed that when using separate thresholds for the two medication states, burst analysis revealed opposite directions of resting drug-related changes between the two sub-beta bands: while the OFF state was associated with more and longer bursts in the low-beta band compared to the ON state (13–20 Hz), less and shorter bursts were observed in the high-beta band (21–30 Hz). These results highlight the distinction between the low- and high-beta bands, and also suggest that previous analysis showing longer beta bursts in the OFF state^16,17,30^ might partly be attributed by the difference in average beta power between the two medication states. Third, using within state normalization, both power and bursts analyses revealed that dopamine medication was associated with increased activities in the alpha and gamma bands around movement execution. This might reflect a loss of frequency specific movement-related modulation in the parkinsonian state, which can be restored with dopaminergic medication, as suggested by a previous study^31^.

Before considering our findings in greater detail, we should stress some of the limitations of our current study. First, recordings were performed in patients with PD in whom recently implanted DBS electrodes had been temporarily externalised. A post-operative stun effect might have therefore influenced the pattern of association between STN LFP activity and motor performance. Second, we should also acknowledge that the number of movements performed in each experimental condition was relatively low, so that weaker correlations may not have been picked up as significant and thereby considered further. Third, it should be stressed that although LFP features in different frequency bands prior and around voluntary movements can predict motor performance, such evidence cannot by itself be taken to establish a causal relationship between the two phenomena. Last but not least, even though we try to compare power and bursting analyses and show the effect of different thresholds in burst analyses in this study, it should be noted that burst and absolute power of the signal are not independent factors but interact in some complicated manner, as the burst duration tends to correlate with amplitude^16^ and more time in bursts will lead to higher average power^13^. Average power and time in bursts were significantly correlated especially when these features were quantified on trial-by-trial basis and over short time periods. Normalising the power within condition before the burst analysing may further differentiate the burst characteristics from the average absolute power, which has not been implemented in this study.

Results of this study highlight the distinction between the low- and high-beta frequency bands. One neurophysiological hallmark of PD is the exaggerated level of beta synchrony observed in the dopamine depleted state, which is commonly characterized by an increase in absolute beta power^32^. As shown here, this excessive activity is also associated with increased average power and a higher probability of bursting in the low beta band, and can be attenuated by levodopa treatment, probably via the restoration of the net dopaminergic activity to its normal range^33^. However, while previous studies have found longer beta bursts in the dopamine depleted state^16,17,30^, the present study suggest that this effect might have been driven by the higher level of average beta power in that state. When this difference in mean power was removed by using separate thresholds for different medication conditions, our results showed that medication modulates burst rate and duration in an opposite way between the low- and high-beta bands. In particular, the number and duration of bursts in the high-beta frequency band increased duration the ON medication state. This speaks in favour of the existence of two functionally distinct beta rhythms in the STN^31,34^. The low-beta rhythm has been found to be more affected by dopaminergic depletion^35,36^, as confirmed by our results showing an increase in power and bursting probability between 10 and 18 Hz. Treatment related suppression of activity in the low-beta band is also more robustly correlated with the improvement of clinical signs of parkinsonism^37,38^, and preferentially supressed by DBS^36,39^. A more recent study showed that beta oscillation frequency is strongly coupled with dopamine tone in both monkeys and humans, with acute dopamine up- and down-modulation resulted in clear shifts up and down the beta frequency domain^40^. All together these findings suggest that the low-beta rhythm may play a pathological ‘antikinetic’ role in the human STN^41^, and is more likely to reflect sustained oscillations in the parkinsonian state. In contrast, activity in the high-beta band is most synchronised with cortical activity^42,43^ and has been suggested to be essentially physiological, being for instance related to force generation^44^. Our results are in line with this interpretation by showing that bursts in the high-beta frequency band are, first, increased by levodopa (rate and duration) which may indicate more effective communication between the STN and the cortex even without changing the average power, and second, associated with motor performance.

When it comes to the prediction of motor performance, both power and bursts analyses revealed a robust and positive relationship between low gamma activity (power and burst rate) around movement onset and movement velocity. This is in keeping with a large body of the literature showing that subcortical gamma activity is involved in the coding of motor parameters, such as the gripping force^6,45^, or the velocity and vigour of voluntary movements^8,10,46^. Using the burst rate as predictor provided complementary information by showing that more high-beta bursts during the preparation period was associated with slower movements in the OFF state. As suggested by previous work, the different features expressed by neural time series (such as the power and bursting activity studied here) might capture different mechanisms or neural functions^29,47^.

Together our results confirmed previous observations that levodopa treatment impacts the oscillatory activity of the basal ganglia circuit beyond the beta range in patients with PD. Methods designed to detect fast changing, transient states such as the Hidden Markov Modelling have for instance shown reduced activities in the theta and low gamma oscillations^35^ in parkinsonian state. Gamma bursts were also modulated by dopaminergic medication with increased duration and bursting rate when ON medication^10^. More generally, it has been suggested that the lack of dopamine determines a loss of segregation between rhythms operating at different frequencies, leading to a pathophysiological information processing in the human basal ganglia^31^. Our results could be interpreted in such a framework. We found that in the OFF state, all frequency ranges showed similar movement-related desynchronization with the power and bursts gradually reducing from rest to movement execution. In contrast, the ON state was characterized by a desynchronization specifically tuned to the beta band and a clear event related synchronization (ERS) in the gamma band. As the gamma ERS seem to be positively associated with motor performance, these results suggest that a clear segregation between the different rhythms might be a critical factor for physiological motor processing.

The ‘burst’ interpretation has far-reaching implications about the nature of neural oscillations. Do the oscillations happen as isolated burst-events or are they sustained phenomena with dynamic amplitude variations, with high power bursting events defined as bursts on top of background tonic oscillations? This may be different for different frequency bands, and even different for different brain states for the same frequency band. For the case of beta oscillation in the healthy sensorimotor cortical-basal ganglia network, single trial analysis of LFPs recorded from striatum and motor-premotor cortex in healthy monkeys showed that brief bursts of oscillation with the duration of 50–150 ms are responsible for virtually all beta-band activity, and that most of the modulations in trial-averaged beta power primarily reflect modulations of burst density^13^. This is consistent with results from healthy human participants showing that high-power beta events from somatosensory and frontal cortex typically lasted < 150 ms and had a stereotypical non-sinusoidal waveform shape^24^. These observations support the hypothesis that physiological beta oscillations in the sensorimotor cortical-basal ganglia network happen as transient, isolated burst-events in healthy normal function, which is better described by their rate, timing, duration, and shape. However, Parkinson’s patients off medication have been shown to exhibit an abnormally large proportion of “long” beta bursts ( > 400 ms)^16^. The averaged beta burst duration, especially in the low beta frequency band, correlated significantly with motor impairment in a large cohort of patients^48^. In addition, Little et al.^47^ reported negative correlation between the temporal variation of beta power and motor impairments, with patients with the most severe motor symptoms showing high average power but low variation in power in the beta band. Therefore, pathological beta oscillations in the parkinsonian state are characterised by higher long-term average power, reduced temporal variation and prolonged beta bursts, indicating that there is component of tonic sustained oscillation, apart from transient high-power events. If the bursts are defined using a single threshold from the pooled data across conditions, difference in the burst characteristics between conditions may be exaggerated^49^, and mainly contributed by the average power difference across conditions. In this case, if bursts are defined using threshold for ON and OFF medication separately, burst analysis will remove the effect of the difference in the tonic activity quantified by average power. Therefore, the power analyses (which takes into account the tonic oscillation) and burst analyses (more suitable for transient burst events) reveal complimentary information on how beta activities are modulated by medication, movements, and how characteristics in beta oscillations predict motor behaviour, as shown in this study.

The nature of the oscillations can also be different for different frequency bands. In this study, we showed that for low gamma activities, bursts analysis revealed the modulation effect of dopamine during rest (30–45 Hz, Figs. 2,3) that was not observed in the averaged power, and the results of the burst analyses are similar for different threshold methods (either separate thresholds for different conditions as shown in Fig. 2f or common threshold across conditions as shown in Fig. 3c). These results suggest that low gamma oscillations are mainly composed of bursting event, with no difference in the tonic activity across medication conditions.

With increasing interest in the ‘bursting’ properties of the beta oscillation, different algorithms have been proposed to identify and quantify bursts^48^. Apart from the threshold-based method, in which a certain percentile of a population of measured power is used to define the bursts, other studies^20,50^ propose to determine beta bursts using a power baseline based on spectral activity that overlapped a simulated 1/f spectrum. However, our recent study show that both high frequency STN stimulation and dopaminergic medication change the 1/f component in the power spectra of the LFPs^51^. Therefore, the baseline method doesn’t get around the question of how to define the ‘baseline power’: either for separate conditions separately, or across pooled conditions. How to define bursts also has practical implications in the threshold-based adaptive deep brain stimulation (aDBS) which uses the beta power as a feedback signal – how shall we define the ‘threshold’ to trigger the DBS? Can we use a fixed threshold pre-defined in a specific medication condition, or shall we consider changing the threshold over time depending on the different medication states? This might have further implication in the optimization of beta-driven DBS algorithms in which both the definition of a burst threshold and the selection of a beta frequency are key steps^52^. Further studies would be required to investigate the clinical implications on therapeutic effect.

## Methods

### Subjects

Ten patients (2 females) with PD gave their written informed consent to participate in the experiment, which was approved by the joint ethics committee of the National Hospital for Neurology and Neurosurgery and the Institute of Neurology, London, and the ethics committee of the Charite ´, Berlin, in accordance with The Code of Ethics of the World Medical Association (Declaration of Helsinki 1967). Their mean age of the participants at the time of the recording was 60.8 years (range 42 to 70 years) with average disease duration of 12.3 years (range 6–18 years). Handedness was assessed using the Edinburgh Handedness Inventory (Oldfield, 1971) and all patients had normal or corrected-to-normal vision.

Clinical severity was measured by using the Unified Parkinson’s Disease Rating Scale and the mean score was 48.9 (standard deviation 15.9; range 24–70) when OFF dopaminergic medication and 20.6 (standard deviation 10.8; range 8–42) in the ON medication state. Patients were implanted with deep brain stimulation (DBS) electrodes simultaneously in the left and right subthalamic nucleus. The DBS electrodes used were model 3389 (Medtronic Neurological Division, Minnesota, USA) with four platinum-iridium cylindrical contacts of 1.27 mm diameter, 1.5 mm length, and 2 mm center-to-center separation. The contacts were numbered 0 (lowermost) to 3 (uppermost). Correct placement of the DBS electrodes was confirmed by intraoperative microelectrode recordings in 8 patients and by postoperative imaging in all patients. Seven cases have been previously published (five as cases 2, 5, 6, 7, and 14 in Doyle et al. 2005 and two as cases 5 and 9 in Fogelson et al. 2005).

### Experimental protocol and data recording

While seated comfortably, subjects were instructed to make rapid stereotyped self-paced movements of a hand-held joystick forward and then immediately backward, repeated approximately every 10–20 s (see Fig. 1a). This action was performed with each hand separately and both in the OFF and ON medication states. Patients were asked to try to keep movements as similar as possible throughout the trials and drug states. The average number of trials performed was 24.1 (range 16–42) per condition with an average inter-movement interval of 15.6 ± 0.74 s (similar delay across conditions, as no significant effect of hand laterality (b = −2.82 ± 2.42, t_(34)_ = −1.16, *p* = 0.25), medication (*b* = −2.98 ± 2.38, t_(34)_ = −1.25, *p* = 0.22), or their interaction (*b* = 2.29 ± 1.5, t_(34)_ = 1.49, *p* = 0.14) were observed when using LME as described in the section ‘statistics’).

Electrophysiological activities in the two STNs were recorded between 1 and 6 days postoperatively, while electrode leads were still externalized and before implantation of the pulse generator. The recordings were made after patients had been off antiparkinsonian medication overnight and again about 1 h after they had taken a minimum of 200 mg levodopa, when the effect of the drug was clinically apparent. Local field potentials (LFPs) were recorded in bipolar differential mode from the adjacent contacts of each DBS electrode (0–1, 1–2, 2–3) to limit volume conduction^53^. An electrode on the forehead was used as ground. Amplification, filtering and recording of LFPs (1–250 or 300 Hz) were performed using a custom-made, high-impedance amplifier (which had at its front end input stage the INA 128 instrumentation amplifier, Texas Instruments) in cases 3 and 7–10, or a D150 amplifier (Digitimer, Welwyn Garden City, Hertfordshire, UK) in cases 1, 4 and 5, before data capture through a 1401 AD converter (Cambridge Electronic Design, Cambridge, UK) onto a portable computer using Spike2 software (Cambridge Electronic Design). In cases 2 and 6, a Schwartzer 34 amplifier system (Schwartzer GmbH, Medical Diagnostic Equipment, Munich, Germany) and Brainlab software (OSG bvba, Rumst, Belgium) were used. Signals were sampled at either 625 Hz or 2 kHz. Joystick movements were simultaneously recorded (DC to 250 Hz) using the same systems and sampling rates.

### Behavioural analysis

Behavioural data were exported and analysed using custom-written MATLAB scripts (version R2019b; MathWorks). Data of the joystick position were band pass filtered between 1 and 100 Hz and interpolated to a common sampling rate of 625 Hz, the lowest original sampling rate used across all patients. The position of the joystick was differentiated to calculate velocity, which was subsequently filtered through a Gaussian kernel with a window duration of 10 ms. As illustrated in Fig. 1b, the joystick velocity profiles were characterized by two distinct peaks corresponding to the forward and backward movements, respectively. Based on the velocity profiles, the movement onset (MO) was defined for each single movement as the first time when the joystick velocity crossed the threshold of three times the standard deviation of the signal (and its noise) at rest, and sustained this speed for at least 100 ms. Continuous signals were then segmented into 9 s trials, from −6 s to +3 s around the MO. The velocity peak of the forward movement was defined for each trial as the maximum in the 700 ms following the MO, and both its amplitude and latency were extracted for each individual trial (VPa and VPt, respectively). Due to the high kinematic variability between and within subjects, the velocity profiles of all individual trials were visually inspected to manually correct movement onset and peak velocity when necessary. For further analyses, trials with less than 6 s after the previous movement were disregarded. Similarly, trials with abnormal hand path trajectories or in which the hand was not maintained stable enough during the inter-trial interval were visually identified and excluded. Finally, the averaged VPa and VPt, i.e. the amplitude and time to reach peak velocity, were computed for each patient and condition.

### STN-LFP pre-processing

All LFP data pre-processing was performed off-line using custom-written MATLAB scripts (version R2019b; MathWorks) and the free and open-source Fieldtrip toolbox^54^. LFP recordings were also band pass filtered between 1 and 100 Hz, interpolated to a common sampling rate of 625 Hz, and segmented into 7 s epochs, from −5 s until 2 s after the MO. Individual trials were visually inspected, and those with channels containing obvious artefacts were excluded from additional processing. After behavioural and electrophysiological artefact removal, analyses were based on averages of 19.3 trials (range 12–31) per subject and condition (4 conditions: OFF-left hand, OFF-right hand, ON-left hand, and ON-right hand). For the rest of the analysis pre-movement period was defined as the 5 s preceding movement onset, in order to avoid any contamination from the execution of the preceding movement.

### STN-LFP analysis: Contact selection

Single-trial LFP signals were transformed in the time-frequency domain by convolution with the complex Morlet’s wavelets characterized by the ratio f0/σf = 7, with f0 ranging from 1 to 50 Hz by steps of 0.25 Hz. For the contact selection, event-related changes in power were calculated by normalizing for each frequency band, the value of each time point against the mean power calculated across all trials. Normalisation limited the effects of different recording systems and of any small variance in targeting. The subject-normalized power was separately averaged over all trials for each of the three bipolar contacts for each STN side. The bipolar contact with the largest power change associated with the contralateral hand movement across both medication states in the whole beta band (13–30 Hz), i.e., the largest difference between the trough of the event-related desynchronization (ERD) during movement and the peak post-movement event-related synchronization (ERS) in the beta band, was then selected for additional analysis. This was motivated by evidence linking maximal beta band activity to the dorsal (motor) region of the STN and to the site that offers the most effective DBS^55^. The contact selection was done for the STN contralateral to the moving hand. Note that only the STN contralateral to movement was considered in the current study. Time-frequency maps and normalized beta power time-courses were also visually inspected to confirm the contact selection.

### LFP analysis: Power spectral analysis

Spectral power were first analysed at rest by computing PSDs using the Welch’s method with a 1 s Hanning window and 50% overlap. The resting period was defined from 5 until 3 s before MO in order to avoid any contamination from LFP activity related to the previous or the following movements, both occurring at least 3 s before or after this period. Power spectra were normalized to the percentage of total power of 4–48 Hz and 52–98 Hz to allow averaging across patients. The 48–52 Hz range was omitted to avoid any contamination from line noise.

Movement-related changes in oscillatory power were evaluated in pre-defined frequency bands for each STN separately (alpha 8–12 Hz, low beta 13–20 Hz, high beta 21–30 Hz, and low gamma 31–48 Hz), averaged across them and then contrasted between the two medication states. To this end, two different normalization were tested (see Fig. 4). First, power time courses were normalized at each time point against the average power across the whole recording sessions of both medication states (OFF and ON). This across-states normalization was used to be able to average all STNs together while keeping the effect of medication, and allow comparison with the common threshold used to define bursts (see below). Second, power time courses were normalized separately within each medication state, to remove the effect of medication and focus on the movement-related modulation. This will be compared with the use of separate burst thresholds.

### LFP analysis: burst detection and features

To determine when a burst occurred, the following steps were applied using custom-written MATLAB scripts (version R2019b; MathWorks). First, power time courses were computed for each single trial and each frequency from 4 to 48 Hz. Two different thresholds were applied to detect burst. In both approaches, thresholds were set at the 75th percentile of the mean beta power calculated for each subject and each STN side over the corresponding frequency across the selected recording condition. In the first approach, a common threshold defined across drug states was applied to data from both drug states as in the previous study^16^. In this case, the 75th percentile of data from both conditions pooled together was used as the threshold for a given hemisphere. The second burst detection threshold was defined for each drug state separately in order to study burst dynamics within drug state and so as to allow direct comparison with previous results where only one drug state has been assessed^21,22^. To this end we separately determined the 75th percentile thresholds from the data recorded ON and OFF medication. In both approaches, all time points surpassing the thresholds were labelled as “potential bursts” and only those lasting more than two oscillatory cycles were definitively defined as “bursts”. Several different approaches have been proposed for the definition of bursts, especially in the beta band^16,17,50^. We opted for the 75th percentile threshold definition so as to allow direct comparison of our results with those of other studies examining medication effect in PD patient^16^ and trial-by-trial effects^21,22^. The burst probability was then defined as the probability of a burst to occur at each time point across all trials.

To better characterize how resting and pre-movement bursting activity relates to motor performance within trial, two additional burst features were extracted in three consecutive time windows; Rest (see above), Pre-Move and Move. The Pre-Move window, defined from 3 to 1 s before MO, was considered to reflect movement preparation, while the Move window was defined around movement execution (from – to +1 around MO). For each time window, the burst rate and duration were extracted. The selection of these two features was based on previous results showing that both might be critical features of the bursting dynamic^16,17,56,57^. Burst duration was defined as the time over which the amplitude remained above threshold, and was averaged between all the bursts detected in the given time window. The burst rate was defined as the number of burst per second. The combined effect of burst rate and duration was also evaluated by computing the time spent in burst, expressed as a percentage.

### Statistical analysis

Statistical analyses were performed using custom-written scripts in MATLAB (R2019b). The modulation of movement velocity across the four conditions was tested by using linear mixed-effect (LME) models with the averaged Velocity Peak amplitude (VPa) and time (VPt) set as dependent variable, and the medication state and hand laterality and their interaction as fixed effects (*fitlme* function, Fig. 1c). Based on the lack of effect of hand laterality, we treated each hand and contralateral STN as an independent sample in the following analysis. The averaged VPa and VPt were then compared between the two medication states using paired samples t-tests (Fig. 1c).

PSDs were compared between the two medication states along the frequency axis (from 4 to 48 Hz) by using cluster-based permutation tests to correct for the multiple tests along the frequency axis using custom-written MATLAB script based on Maris & Oostenveld, 2007^58^ (Figs. 2b, d and 3b). Cluster-based permutation tests were also used to evaluate whether medication significantly affects the burst features (burst duration and rate) from 4 to 48 Hz (correction along the frequency axis, Figs. 2e and 3c), and the level of normalized power (Fig. 4) and burst probability (Fig. 5) over time.

Linear mixed effect models were used to test how averaged burst rate and duration were modulated by medication when approaching movement (Fig. 6). To this end, averaged burst rate and duration were calculated for each pre-defined frequency bands and were entered as dependant variables in the model (one model tested per frequency band). The time windows (rest, pre-move, move) and medication states (ON, OFF) were entered as fixed-effects and their interaction were tested. Nested models were compared with likelihood ratio tests, to assess whether the model’s improved fit to the data merited the added complexity associated with the inclusion of the interaction component. If the interaction was not significant, a model without interaction was considered. If the interaction was significant (reported as Time * Med in Fig. 6), follow-up LME models were run separately within each medication state, with only the burst feature as fixed effect.

The single-trial relationship between pre-movement LFP activity and behavioural performances was assessed through linear mixed-effect (LME) models applied to single trials. The velocity peak of each single trial was set as dependent variable. To correct for the non-normality of the dependent variable, the peak velocities were raised by the λ exponents identified by a Box-Cox procedure (power transformation). Then LME models were tested for each frequency (from 8 to 48 Hz) and each time-windows separately. Medication state (OFF or ON) and average power or time spent in burst at the specific frequency were entered into the model as fixed-effects and their interaction were tested. All models were estimated by the method of maximum likelihood and included random intercept grouped by subjects to capture individual differences. Residuals plots of every model were visually inspected to control for any obvious deviation from homoscedasticity or normality. Finally, the p-values of the LME models were corrected for the multiple comparisons along the frequency axis using the false discovery rate procedure.

### Reporting Summary

Further information on research design is available in the Nature Research Reporting Summary linked to this article.

## Supplementary information

Supplementary material

Reporting summary checklist

## Data Availability

The electrophysiology dataset is not yet openly available, as participants only agreed for the data to be used in specified studies at the time of recording. We welcome enquires for sharing this as part of a collaboration, please contact andrea.kuehn@charite.de or huiling.tan@ndcn.ox.ac.uk

## Code availability

Custom-written Matlab scripts for cluster-based permutation tests have been used in previous studies, and can be find in the following link: 10.5287/bodleian:M81wpxae8.
