# Supplementary material for: Average power and burst analysis revealed complementary information on drug-related changes of motor performance in Parkinson’s disease
Source: NPJ Parkinsons Dis. 2023 Jun 16;9:93. doi: 10.1038/s41531-023-00540-x (PMC10275865; doi:10.1038/s41531-023-00540-x)
Supplement: Supplementary file 1 — Supplementary material [file 41531_2023_540_MOESM1_ESM.pdf]

Supplementary Material

1. Model 1: VP ~ BurstTime+ AbsolutePower + Medication + (1|Subject)

BurstTime: time spent in burst in the Move window (-1 to 1 second around movement onset)
AbsolutePower: absolute power in the low gamma band during the Move window (-1 to 1 second around movement onset)

Model Results:

Name		Estimate	SE	tStat	DF	pValue
Intercept		0.096	0.011	8.42	691	<0.001
Absolute Power		-0.12	0.047	-2.55	691	0.01
BurstTime		0.04	0.010	3.90	691	<0.001
Medication		0.022	0.002	11.4	691	<0.001

The model showed that both absolute power and the 'time in burst' in the low gamma frequency band had independent prediction power for peak velocity. However, the effect of the absolute power and time in burst is opposite, with positive estimated slope for the effect of 'time in burst' and negative slope for the effect of 'average power'. We think this is because the 'average absolute power' and the 'time in burst' is highly correlated, as confirmed by relative large value (5.18) for variance inflation factor (VIF) between the two variables. Therefore, it would be very difficult to disambiguate the effect, and results of linear model with two highly correlated independent variables might not be valid and difficult to interpret.

2. Model 2: VP ~ Burst*Med+ BurstTime + AbsolutePower + Medication + (1| Subject)

BurstTime: time spent in high beta burst in the Move window
AbsolutePower: absolute power in the high beta band during the Move window

Model Results:

Name		Estimate	SE	tStat	DF	pValue
{'(Intercept)'}		0.11	0.012	8.90	690	*>0.001*
Absolute Power		-0.02	0.016	-1.03	690	0.30
Medication		0.019	0.003	6.79	690	>0.001
Burst time		-0.08	0.035	-2.40	690	0.016
Med:Burst interaction		0.05	0.020	2.50	690	0.013

The result of Model 2 suggest that compared with absolute power, time in burst for high beta band contains more information in predicting peak velocity, with longer time in burst in medication OFF state predicted lower peak velocity. However, when the data from different medication conditions were considered separately, the effects were only significant for the ON med conditions:

3. Model 3: VP ~ BurstTime + AbsolutePower + (1| Subject) (for high beta band activities, and ON meds only)

Model Results:

Name		Estimate	SE	tStat	DF	pValue
Absolute Power		0.053116	0.020978	2.532	369	0.011757
Burst time		-0.02754	0.011598	-2.375	369	0.018082

As in Model 1, effects of the ‘absolute power’ and time in burst had opposite directions. Again, we think this is because the ‘time in burst’ and ‘absolute power’ are highly correlated, as confirmed by a large variance inflation factor (6.47) between the two variables in the high beta band. The collinearity between the intendent variables make the model less reliable and more difficult to interpret.

Given the results above, it is difficult to disambiguate the effect of power vs. bursts, especially when the average power were quantified during short time windows on trial-by-trial basis.
