# Supplementary material for: Average power and burst analysis revealed complementary information on drug-related changes of motor performance in Parkinson’s disease
Source: NPJ Parkinsons Dis. 2023 Jun 16;9:93. doi: 10.1038/s41531-023-00540-x (PMC10275865; doi:10.1038/s41531-023-00540-x)
Supplement: Supplementary file 2 — Reporting summary checklist [file 41531_2023_540_MOESM2_ESM.pdf]

# nature portfolio

Corresponding author(s): Prof. Huiling Tan
NPJPARKD-01589

Last updated by author(s): Sep 16, 2022

# Reporting Summary

Nature Portfolio wishes to improve the reproducibility of the work that we publish. This form provides structure for consistency and transparency in reporting. For further information on Nature Portfolio policies, see our Editorial Policies and the Editorial Policy Checklist.

## Statistics

For all statistical analyses, confirm that the following items are present in the figure legend, table legend, main text, or Methods section.

| n/a | Confirmed | |
|---|---|---|
| ☐ | ☒ | The exact sample size (*n*) for each experimental group/condition, given as a discrete number and unit of measurement |
| ☐ | ☒ | A statement on whether measurements were taken from distinct samples or whether the same sample was measured repeatedly |
| ☐ | ☒ | The statistical test(s) used AND whether they are one- or two-sided<br>*Only common tests should be described solely by name; describe more complex techniques in the Methods section.* |
| ☐ | ☒ | A description of all covariates tested |
| ☐ | ☒ | A description of any assumptions or corrections, such as tests of normality and adjustment for multiple comparisons |
| ☐ | ☒ | A full description of the statistical parameters including central tendency (e.g. means) or other basic estimates (e.g. regression coefficient) AND variation (e.g. standard deviation) or associated estimates of uncertainty (e.g. confidence intervals) |
| ☐ | ☒ | For null hypothesis testing, the test statistic (e.g. *F*, *t*, *r*) with confidence intervals, effect sizes, degrees of freedom and *P* value noted<br>*Give P values as exact values whenever suitable.* |
| ☒ | ☐ | For Bayesian analysis, information on the choice of priors and Markov chain Monte Carlo settings |
| ☒ | ☐ | For hierarchical and complex designs, identification of the appropriate level for tests and full reporting of outcomes |
| ☒ | ☐ | Estimates of effect sizes (e.g. Cohen's *d*, Pearson's *r*), indicating how they were calculated |

*Our web collection on statistics for biologists contains articles on many of the points above.*

## Software and code

Policy information about availability of computer code

| | |
|---|---|
| Data collection | Data collection was performed using custom-written software. |
| Data analysis | All analysis was done in MATLAB using custom-written codes that are available from the corresponding author upon reasonable request. |

For manuscripts utilizing custom algorithms or software that are central to the research but not yet described in published literature, software must be made available to editors and reviewers. We strongly encourage code deposition in a community repository (e.g. GitHub). See the Nature Portfolio guidelines for submitting code & software for further information.

## Data

Policy information about availability of data

All manuscripts must include a data availability statement. This statement should provide the following information, where applicable:

- Accession codes, unique identifiers, or web links for publicly available datasets
- A description of any restrictions on data availability
- For clinical datasets or third party data, please ensure that the statement adheres to our policy

All data will be uploaded to the Medical Research Council Brain Networks Dynamics Unit (MRC BNDU) Data Sharing Platform at the University of Oxford.

## Human research participants

Policy information about studies involving human research participants and Sex and Gender in Research.

| | |
|---|---|
| Reporting on sex and gender | Data from 8 males and 2 female Parkinson's disease patients were included. |
| Population characteristics | Parkinson's patients that were included in our study were diagnosed on average 12.3 (6-18 years) years before they participated and were on average 60.8 years old (42 to 70 years) |
| Recruitment | Patients that were eligible for inclusion were diagnosed with Parkinson's disease and at an advanced stage of the disease. They were therefore selected and consented for deep brain stimulation surgery. Due to this, we only studied Parkinson's patients at an advanced stage. Recruitment took place in two different centres: Institute of Neurology, London, and the neurology department of the Charité, Berlin |
| Ethics oversight | This protocol was approved by the joint ethics committee of the National Hospital for Neurology and Neurosurgery and the Institute of Neurology, London, and the ethics committee of the Charité, Berlin, in accordance with The Code of Ethics of the World Medical Association. |

Note that full information on the approval of the study protocol must also be provided in the manuscript.

# Field-specific reporting

Please select the one below that is the best fit for your research. If you are not sure, read the appropriate sections before making your selection.

☒ Life sciences ☐ Behavioural & social sciences ☐ Ecological, evolutionary & environmental sciences

For a reference copy of the document with all sections, see nature.com/documents/nr-reporting-summary-flat.pdf

# Life sciences study design

All studies must disclose on these points even when the disclosure is negative.

| | |
|---|---|
| Sample size | In keeping with an abundance of literature on human electrophysiology, we did not predetermine the sample size. As the recordings of externalized DBS patient is limited we took all the human data that was available to us for statistical analysis. In doing so, our human sample is in line with most studies investigating local field potentials from the subthalamic nucleus in Parkinson's disease (Little et al., 2013; Kuhn et al., 2006; Swann et al., 2016 and many more). |
| Data exclusions | We only kept the dataset when patients performed the same motor task in both medication state (OFF and ON). We also removed trials with movement artifacts or line inference as described in the manuscript. |
| Replication | We started our analysis by the replication of a well known findings: the increase in beta power OFF medication in patient with PD. This replication allowed us to then explore the modulation of the LFP activity further. |
| Randomization | We did not have a randomisation step. All human participants were treated equally and underwent the same protocol. |
| Blinding | There was not group allocation as all patients performed the tasks in the two conditions (OFF and ON). Note however that the investigator analyzing the data did not participate to the recordings and vice versa. |

# Reporting for specific materials, systems and methods

We require information from authors about some types of materials, experimental systems and methods used in many studies. Here, indicate whether each material, system or method listed is relevant to your study. If you are not sure if a list item applies to your research, read the appropriate section before selecting a response.

## Materials & experimental systems

| n/a | Involved in the study | |
|---|---|---|
| ☒ | ☐ | Antibodies |
| ☒ | ☐ | Eukaryotic cell lines |
| ☒ | ☐ | Palaeontology and archaeology |
| ☒ | ☐ | Animals and other organisms |
| ☐ | ☒ | Clinical data |
| ☒ | ☐ | Dual use research of concern |

## Methods

| n/a | Involved in the study | |
|---|---|---|
| ☒ | ☐ | ChIP-seq |
| ☒ | ☐ | Flow cytometry |
| ☒ | ☐ | MRI-based neuroimaging |

## Clinical data

Policy information about clinical studies

All manuscripts should comply with the ICMJE guidelines for publication of clinical research and a completed CONSORT checklist must be included with all submissions.

| | |
|---|---|
| Clinical trial registration | Our study does not include a blinded, randomised clinical trial. For this reason, we did not stick to the CONSORT criteria when reporting our results. |
| Study protocol | Our study does not pertain to the criteria of a RCT and was not registered at clinicaltrials.gov or the ISRCTN registry. |
| Data collection | Data collection at the Insitute of Neurology, London and the Hospital la Charité in Berlin. Data collection happened from 2002 to 2004. Data already included in Doyle et al, 2005 and Fogelson et al., 2005. For more details, see the methods section of our manuscript. |
| Outcomes | Our primary outcome was to compare in the same study two different way of analysis LFP data from PD patient to evaluate if they can provide similar or complementary information about how LPF are related to motor performance. To do so we analyse both the average power and burst properties of the LFP following similar steps.<br>Our secondary objective was to evaluate the influence of medication on the LFP-behaviour relationship. Here again we compared the infleucne of medication on both the average power and the burst properties of the LFP signal. |
